# Dorsal Root Ganglion Stimulation for the Management of Phantom Bladder Pain: A Case Report

**DOI:** 10.7759/cureus.55043

**Published:** 2024-02-27

**Authors:** Nirguna Thalla, Isaiah Levy, Anne P Pribonic, Gaurav Chauhan, Suresh K Srinivasan

**Affiliations:** 1 Anesthesiology and Perioperative Medicine, University of Pittsburgh Medical Center, Pittsburgh, USA; 2 Anesthesiology and Perioperative Medicine, University of Pittsburgh Medical Center Presbyterian, Pittsburgh, USA; 3 Pain Management, Trinity West Medical Center, Steubenville, USA

**Keywords:** visceral pain, sacral neuromodulation, dorsal root ganglion stimulation, phantom pain syndrome, phantom bladder pain

## Abstract

Phantom bladder pain, a rare condition following cystectomy, can pose a challenge to pain management providers. We present the case of a 43-year-old male who developed severe phantom bladder pain post-cystectomy. Despite multiple treatments, his symptoms persisted, significantly affecting his quality of life. Dorsal root ganglion stimulation (DRGS) was attempted after conventional therapies failed. The DRGS trial provided significant relief, leading to permanent implantation and a 90% reduction in pain. This case highlights DRGS as a potential treatment for phantom bladder pain, expanding its applications beyond traditional uses. Further research is needed to elucidate its mechanisms and broader applicability.

## Introduction

Phantom bladder pain following cystectomy is a neuropathic pain that occurs after bladder removal surgery. Although phantom limb pain has been well described, other phantom pains are less commonly reported. The etiology of phantom pain is still poorly understood; the predominant theory is that the pain stems from irritation of severed nerve endings, which is supported by the development of neuromas in patients with phantom limb pain, but likely involves many other peripheral and central nervous system factors. Outside of limb pain, the most commonly described phantom pains include phantom breast pain following mastectomy, as well as anal pain following anal resection due to cancer [1,2]. Phantom pain, however, can occur in almost any organ. It often has a neuropathic component, with characteristics such as burning, pins and needles, and stinging. It can be difficult to control with medication management alone, although commonly neuropathic agents including gabapentin and amitriptyline are trialed. To our knowledge, phantom bladder pain has only been described in case reports. No other epidemiological data exists. It involves feeling pain in the area where the bladder used to be, despite its absence. Presumably, like the etiology of other phantom pains, the cause of phantom bladder pain is related to changes in neuronal mechanisms [3]. Phantom bladder pain can present as a sensation of a full bladder, lower abdominal pain, or bladder spasms. This pain can vary in intensity and may require specialized pain management approaches, such as multimodal medication management, nerve blocks (most commonly described are sympathetic ganglion blocks) or neuromodulation techniques, for relief. These treatment measures are commonly explored for other phantom pain conditions. In terms of neuromodulation methods, dorsal root ganglion stimulation (DRGS) has been utilized for phantom limb pain [4,5] but to date there are no published studies regarding its use for phantom pain of other organs. In this report, we present a novel case of phantom bladder pain successfully treated by dorsal root ganglion stimulation. This report serves as support for the potential use of DRGS to treat refractory phantom bladder pain as well as consideration for use in similar pain conditions.

## Case presentation

The patient was a 43-year-old male who presented to the chronic pain clinic for the evaluation of phantom bladder painful spasms. He was referred by a urological surgery specialist for chronic pain management. The patient had a congenital diagnosis of medullary sponge kidney, which involved a multiyear history of urological and renal sequelae starting in his early twenties. He was initially referred to nephrology and urology services due to recurrent nephrolithiasis and nephrocalcinosis. The patient was diagnosed with advanced chronic kidney disease due to recurrent obstructions. Urological surgery involvement resulted in lithotripsy and stenting procedures repeated many times over several years. Disease progression led to recurrent bladder and kidney infections, urethral strictures, and urine output dependent on nephrostomy tubes. Shared decision-making with the patient's treatment team ultimately decided that surgical management was necessary due to the progression of kidney disease to stage 5, multiple life-threatening infection-related hospitalizations, severe lower urinary tract strictures, and the high recurrence rate of obstructions. The patient ultimately underwent bilateral nephrectomy and simple cystectomy with the intent of preparing for a kidney transplant. He was transitioned to dialysis while awaiting his transplant procedure.

The patient first experienced phantom bladder pain several days following the nephrectomy and cystectomy procedure. He described the symptoms as intense, intermittent sharp, spasm-like sensations accompanied by the sensation that he needed to urinate. These symptoms were initially attributed to post-operative pain but remained persistent over time. He did not experience any pain between spasm episodes. The patient did not encounter any postoperative complications, and surgical sites eventually healed without any discernible skin or muscular pathologies. The frequency and duration of his symptoms did not show consistent patterns. The patient had many "bad days" per week that involved over 10 episodes with spasm sensations lasting from 1 to 10 minutes. The painful sensations were rated between 5 and 8/10 on a numeric rating scale (NRS). On occasion, the sensations would wake him up at night. He endorsed a significant impact on his quality of life, affecting his mood, sleep, and social interactions.

Initial treatment aimed to control his phantom urinary spasms/sensations. The patient self-treated with over-the-counter medications such as ibuprofen 800 mg total daily dose, acetaminophen 2000 mg total daily dose, and heat/ice applications to the lower abdominal region. However, he experienced no improvement, so he was prescribed a combination of gabapentin 100 mg daily and amitriptyline 10 mg before bedtime. He was given an additional 100 mg of gabapentin on dialysis days. Unfortunately, he did not experience any improvement in his symptoms with this regimen. He successfully underwent a living donor kidney transplantation with an ileal conduit diversion. He tolerated the procedure and achieved adequate postoperative pain control. However, he continued to experience painful phantom bladder spasms/sensations. Following an improvement in his renal function, his gabapentin and amitriptyline doses were increased to 300 mg three times a day and 75 mg, respectively. Baclofen 10 mg was started and titrated to three times a day dosing. The patient continued to experience little to no relief in both the severity and frequency of episodes. He was changed to several other skeletal muscle relaxants, anticonvulsants such as pregabalin, serotonin noradrenergic reuptake inhibitors (SNRIs) such as duloxetine, and N-methyl-D-aspartate (NMDA) antagonists such as memantine. Maximal doses taken by the patient for each of these medications were pregabalin 100 mg three times daily, duloxetine 60 mg daily, methocarbamol 750 mg three times daily, cyclobenzaprine 10 mg three times daily, and memantine 50 mg daily. The patient also completed a 12-week chronic pain rehabilitation regimen with occupational and physical therapists focusing on desensitization therapies and reducing centralized pain processing. Interventional treatments such as sympathetic blocks to the hypogastric plexus were considered but denied by the patient. None of the medication and rehabilitative approaches provided any additional relief. The patient developed tremors attributed to polypharmacy and was weaned off all pain medications.

A dorsal root ganglion stimulation (DRGS) trial was considered following the failure of multiple conservative measures, poor quality of life, and the duration of symptoms. After a psychological evaluation for untreated mental illness was cleared, the patient consented to the procedure. Initially, the trial was conducted at the S2 neural foramen bilaterally with suboptimal paresthesia coverage. The trial was then conducted with lead placement at the S3 neural foramen bilaterally, resulting in adequate paresthesia coverage of the bladder region according to the patient's live verbal feedback during the trial. He reported more than a 90% improvement in his pain from phantom bladder sensations and also reported a significant (90%) decrease in the frequency of spasms at one week post-trial. He elected to move forward with a permanent implant using a Proclaim DRG system in the bilateral S3 neural foramen (Abbott Laboratories, Abbott Park, Illinois, USA).

**Figure 1:**
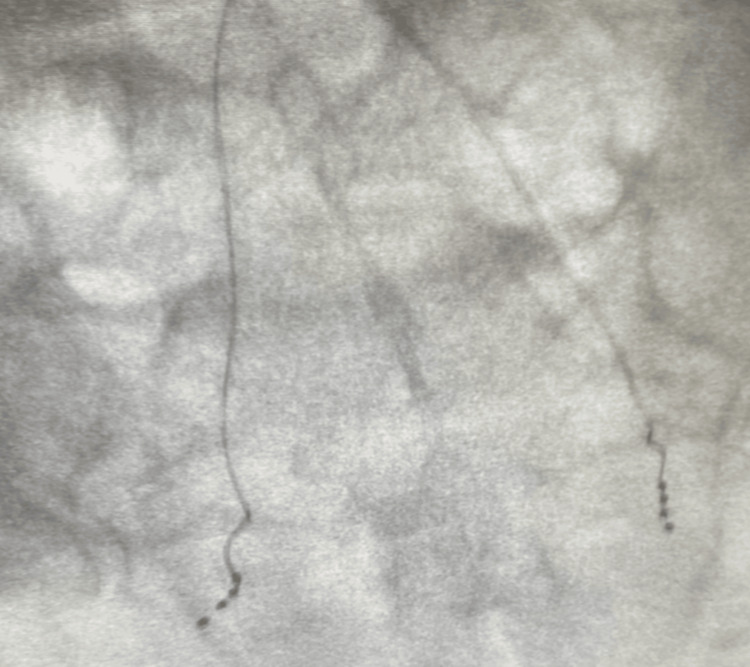
Anteroposterior fluoroscopic view of S3 neuroforaminal dorsal root ganglion lead stimulator placement.

**Figure 2:**
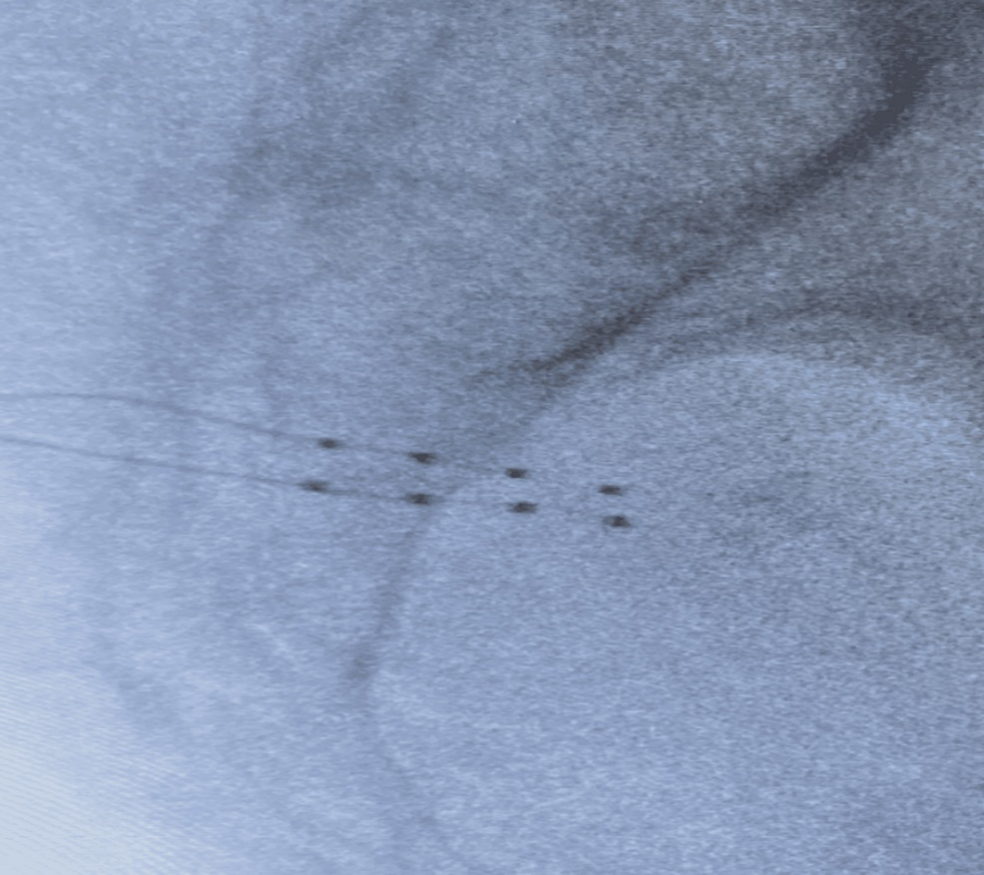
Lateral fluoroscopic view of S3 neuroforaminal dorsal root ganglion lead stimulator placement.

Under fluoroscopy, attention was directed to the right S3 neural foramen. The skin over the right S3 foramen was localized with a 5 ml solution consisting of 10 ml of 2% lidocaine and 10 ml of bupivacaine 0.25%. A Chiba needle was used to locate the right S3 foramen, and then a 14-gauge Tuohy needle was advanced into the right S3 foraminal space. Needle placement was confirmed in AP and lateral views. A guide wire was advanced through the DRG sheath and the Tuohy needle into the appropriate foramen. The guide wire was removed, leaving the lead inserted in the appropriate foramen, next to the DRG. The sheath was adjusted to allow for superior loops to form in the extraforaminal space. The stylet, sheath, and Tuohy needle were removed, leaving only the lead in place. A similar process was repeated on the left S3 foramen (Figure 1 and Figure 2).

A midline incision was made after the instillation of 10 cc of 0.5% bupivacaine in the skin and subcutaneous tissue. The tunneling device was used to transfer the DRG leads to the midline cavity, where they were secured using the anchor devices. The anchors were sutured to the superficial fascia using 2-0 silk sutures. Then the battery site was identified on the right flank, and 0.5% bupivacaine was administered for local anesthesia. A 5 cm incision was made, and a subcutaneous pocket was created. The implantable pulse generator (IPG) was inserted into the pocket after connecting it to the two IPG leads. All impedances were within normal limits. The subcutaneous tissues were sutured with 2-0 Vicryl, and the epidermis was sutured with 3-0 Vicryl. The dressing was applied appropriately.

No unwanted complications were observed during the procedure, and the patient was discharged home from the outpatient surgery department in a stable condition.

At six months following the DRG implantation, he continued to report a 90% improvement in his pain and a 90% reduction in the frequency of spasm/sensations. He is satisfied with the results and is no longer taking any pain medications, reporting an overall improvement in his quality of life.

## Discussion

Spinal neuromodulation has proven to be an effective strategy in managing chronic pain disorders and improving functional outcomes, enhancing the quality of life, and reducing the burden of analgesic medication. The dorsal root ganglion (DRG), located in the intervertebral foramen, receives afferent nociceptive input from primary sensory neurons in the periphery. The DRG is hypothesized to play a vital role in processing, modulating, and maintaining chronic pain signals [6]. DRGS is a spinal neuromodulation technique designed to target DRG neurons and is considered superior to conventional treatment for difficult-to-treat pain diagnoses such as complex regional pain syndrome [7]. DRGS is reported to be effective for chronic pelvic pain, including pain secondary to neurectomy of ilioinguinal, iliohypogastric, and genitofemoral nerves [8,9]. While the precise mechanism(s) of DRGS remains to be elucidated, it is hypothesized that DRGS suppresses neuropathic pain by decreasing the neuronal excitability of the DRG cells. A growing body of evidence suggests DRGS may be a more effective treatment for focal body regions, such as bladder pain syndrome/interstitial cystitis, than traditional dorsal column stimulation. This rationale was applied to make DRGS the first choice neuromodulation modality for this case [8].

Bladder afferent pathways involve the pelvic nerve (parasympathetic, S2-4), hypogastric nerve (sympathetic, L1-L3), and pudendal nerve (somatic, S3) [10]. Sacral neuromodulation targeting the S2-S4 DRG has been used for conditions such as interstitial cystitis, bladder pain syndrome, pudendal neuralgia, coccydynia, and various chronic pelvic pain syndromes [11].

Urinary urgency and pelvic pain have been documented in patients after cystectomy, hemodialysis, or spinal cord injury. These symptoms include lower abdominal pain during urination, frequent urination, a feeling of fullness with a full bladder, and sharp, burning pain during urination [12]. Previous case reports have shown successful treatment of phantom bladder pain and spasms with lumbar sympathetic blocks [13].

Phantom bladder pain involves supraspinal, spinal, and peripheral mechanisms. In phantom bladder pain, DRG mechanisms are thought to include injured residual axons within the DRG and remaining peripheral nerves with spontaneous activity from hyperexcitable loci. This leads to aberrant signaling through the spinothalamic tract, resulting in perceived pain [14]. Previously, DRGS was assessed in case reports and series for chronic pelvic pain, with 15 out of 25 patients across five studies experiencing satisfactory pain relief and significant improvements in quality of life [15]. However, no prior studies to the authors' knowledge, have assessed the utility of DRGS for a phantom visceral/organ pain condition.

## Conclusions

In conclusion, this case report highlights the potential of dorsal root ganglion stimulation as a promising and effective treatment option for the rare and challenging condition of phantom bladder pain following cystectomy. Despite the patient's complex medical history and prior unsuccessful treatments, DRGS provided significant relief from pain and spasms. The patient's improved quality of life following DRGS underscores its potential as a valuable intervention for refractory pain conditions, expanding its utility beyond conventional indications. This success emphasizes the need for further research and exploration of DRGS in managing complex pain disorders.
